# Branched-Chain Amino Acid Supplementation Alters the Abundance of Mechanistic Target of Rapamycin and Insulin Signaling Proteins in Subcutaneous Adipose Explants from Lactating Holstein Cows

**DOI:** 10.3390/ani11092714

**Published:** 2021-09-17

**Authors:** Yusheng Liang, Fabiana F. Cardoso, Claudia Parys, Felipe C. Cardoso, Juan J. Loor

**Affiliations:** 1 Department of Animal Sciences, Division of Nutritional Sciences, University of Illinois, Urbana, IL 61801, USA; yusheng4@illinois.edu (Y.L.); fabiana2@illinois.edu (F.F.C.); cardoso2@illinois.edu (F.C.C.); 2 Evonik Operations GmbH|Nutrition & Care, 63457 Hanau, Germany; claudia.parys@evonik.com

**Keywords:** leucine, adipose tissue, insulin signaling

## Abstract

**Simple Summary:**

Branched-chain amino acids (BCAAs) are import regulators of mechanistic target of rapamycin (mTOR). In humans and rodents, increased circulating BCAA levels are positively associated with changes in protein abundance of insulin and amino acid (AA) signaling pathways in organs such as skeletal muscle and adipose. Unlike aspects of fatty acid metabolism (e.g., lipolysis, lipogenesis), it is unknown if BCAA directly affect subcutaneous adipose tissue (SAT) AA metabolism and insulin signaling. We propose that BCAA availability within SAT could enhance aspects of AA and insulin function by promoting increases in the abundance of key proteins.

**Abstract:**

The objective of this study was to investigate changes in protein abundance of mTOR and insulin signaling pathway components along with amino acid (AA) transporters in bovine s.c. adipose (SAT) explants in response to increased supply of Leu, Ile, or Val. Explants of SAT from four lactating Holstein cows were incubated with high-glucose serum-free DMEM, to which the 10 essential AAs were added to create the following treatments: ideal mix of essential AA (IPAA; Lys:Met 2.9:1; Lys:Thr 1.8:1; Lys:His 2.38:1; Lys:Val 1.23:1; Lys:Ile 1.45:1; Lys:Leu 0.85:1; Lys:Arg 2.08:1) or IPAA supplemented with Ile, Val, or Leu to achieve a Lys:Ile of 1.29:1 (incIle), Lys:Val 1.12:1 (incVal), or Lys:Leu (incLeu) 0.78:1 for 4 h. Compared with IPAA, incLeu or incIle led to greater activation of protein kinase B (AKT; p-AKT/total AKT) and mTOR (p-mTOR/total mTOR). Total EAA in media averaged 7.8 ± 0.06 mmol/L across treatments. Incubation with incLeu, incIle, or incVal led to greater protein abundance of solute carrier family 38 member 1 (SLC38A1), a Gln transporter, and the BCAA catabolism enzyme branched-chain α-keto acid dehydrogenase kinase (BCKDK) compared with IPAA. Activation of eukaryotic elongation factor 2 (eEF2; p-eEF2/total eEF2) was also greater in response to incLeu, incIle, or incVal. Furthermore, compared with incLeu or incIle, incVal supplementation led to greater abundance of SLC38A1 and BCKDK. BCKDK is a rate-limiting enzyme regulating BCAA catabolism via inactivation and phosphorylation of the BCKD complex. Overall, data suggested that enhanced individual supplementation of BCAA activates mTOR and insulin signaling in SAT. Increased AA transport into tissue and lower BCAA catabolism could be part of the mechanism driving these responses. The potential practical applications for enhancing post-ruminal supply of BCAA via feeding in rumen-protected form support in vivo studies to ascertain the role of these AAs on adipose tissue biology.

## 1. Introduction

It is well-recognized in a number of mammalian species that mechanistic target of rapamycin (mTOR), including two distinct protein complexes, mTOR complex 1 (mTORC1) and mTOR complex 2 (mTORC2), regulates protein synthesis, cell growth, and proliferation [1,2,3]. Both complexes are partly controlled by the supply of nutrients such as branched-chain amino acids (BCAA; Leu, Ile, and Val), underscoring the unique role that dietary compounds can have in essential cellular processes.

Supplementation of Val, Leu, and Ile individually to immortalized bovine mammary epithelial cells or bovine mammary tissue slices in vitro (total AA supply averaging 7.2 to 7.8 mmol/L) promoted protein synthesis via upregulating the phosphorylation status of mTOR [4,5]. In an in vivo study, compared with jugular infusion of Met and Lys alone, infusion of Met and Lys plus BCAA did not improve milk protein yield or protein content in high-producing cows [6]; however, adding BCAA to the mix of Met and Lys reduced the milk urea nitrogen content, suggesting that BCAA might affect protein synthesis or degradation in peripheral tissues.

The recent demonstration via metabolomics analysis that a high body condition score is associated with greater BCAA degradation before calving [7] suggested there is a potential relationship between body fat and BCAA metabolism in dairy cows. Although bovine adipose tissue has not been generally considered responsive to AA supply, recent research indicated that, compared with liver and skeletal muscle, the s.c. adipose tissue (SAT) in dairy cows has the greatest mRNA abundance of mitochondrial branched-chain aminotransferase (*BCAT2*) [8]; furthermore, BCAA, Gln, and neutral AA transporters are expressed in bovine SAT [8,9]. Hence, available data support the notion that bovine SAT is a potential site for BCAA uptake and metabolism.

A recent transcriptome analysis of SAT from our laboratory revealed that the BCAA catabolism pathway and upstream regulators of cytokines were both inhibited after parturition, underscoring the physiological relevance of BCAA metabolism in regulating adipose function [10]. Whether BCAA contribute directly to activation of mTOR or the abundance of proteins related with insulin and amino acid signaling in bovine SAT is unclear. Thus, the primary objective of this study was to determine the in vitro effects of enhanced BCAA supplementation on protein abundance of key components of the mTOR and insulin signaling pathway in bovine SAT explants.

## 2. Methods

### 2.1. Cows

All procedures were approved by the University of Illinois Institutional Animal Care and Use Committee (Urbana; protocol#19036). Four clinically healthy multiparous lactating Holstein cows from the University of Illinois dairy herd were used. Average parity, body weight, days in milk, and milk yield prior to slaughter were 4 ± 0.4, 696 ± 20.4 kg, 248 ± 18, and 27.0 ± 3.5 kg/day (mean ± SD), respectively. Cows were fed the same diet formulated according to NRC [11] once daily. Ingredients and nutrient composition of the diet are reported in Supplemental Tables S1 and S2. All cows were milked twice daily, housed in a free-stall barn, and had free access to water.

### 2.2. Tissue Collection, Processing, and Cell Culture

Cows were euthanized by captive bolt at the College of Veterinary Medicine diagnostic laboratory facilities (University of Illinois, Urbana, IL, USA). Subcutaneous adipose tissue samples from the tail-head were obtained immediately post-slaughter and, within 30 min of collection, brought to the laboratory in warm Dulbecco’s modified Eagle’s medium and Ham’s F-12 nutrient mixture (DMEM:F-12; Sigma-Aldrich, St. Louis, MO, USA) containing 1% penicillin/streptomycin (Pen/Streptomycin; Sigma-Aldrich, St Louis, MO, USA). Prior to culture, SAT was trimmed into pieces using a sterile scalpel blade in a sterile petri dish (catalog No. 101VR20, Thermo Fisher Scientific, Waltham, MA, USA), and then 200 mg tissue was incubated in duplicate in 5 mL of medium in six-well plates. Our previous study with bovine mammary epithelial cells provided evidence that increasing the BCAA to Lys ratio could promote milk protein synthesis via enhancing the activity of mTORC1 and upregulating mRNA abundance of AA transporters [10]. Culture media were as follows: ideal profile of essential AA (EAA) as the control (IPAA), increased Leu (incLeu; Lys:Leu 0.78:1), increased Ile (incIle; Lys:Ile 1.29:1), or increased Val (incVal; Lys:Val 1.12:1) (Table 1). The concept of “ideal” AA ratios in the context of milk protein synthesis has been explored across a number of in vivo and in vitro experiments, and our previous in vitro work with bovine mammary cells [10] served as the basis for the present study. Furthermore, the ideal AA ratio concept also was discussed in the NRC [5] and by other research groups [11].

The 10 EAA (L-isomer, Sigma-Aldrich, St Louis, MO, USA) were added into the custom high-glucose serum-free DMEM (devoid of these 10 EAA, custom made from Gibco, Carlsbad, CA, USA). Media were prepared according to previous studies from our group [5,12]. Briefly, the formulation of the essential AA was as follows: control medium with the ideal AA ratio (IPAA, Lys:Met 2.9:1; Lys:Thr 1.8:1; Lys:His 2.38:1; Lys:Val 1.23:1; Lys:Ile 1.45:1; Lys:Leu 0.85:1; Lys:Arg 2.08:1), incLeu (Lys:Leu 0.78:1), incIle (Lys:Ile 1.29:1), and incVal (Lys:Val 1.12:1). Media were prepared by increasing Leu, Ile, or Val, individually, while maintaining other AA ratios the same as in IPAA. Subcutaneous adipose explants were incubated in a humidified incubator at 37 °C with 5% CO_2_. After 4 h incubation, two SAT explants per treatment were transferred from six-well plates to screw-capped microcentrifuge tubes, snap frozen in liquid nitrogen, and stored at −80 °C until further analysis.

### 2.3. Western Blotting

Total protein was extracted using RIPA Lysis and Extraction Buffer (catalog No. 89900, Thermo Fisher Scientific, Waltham, MA, USA) following the manufacturer’s protocols. Protein concentration was determined using the Pierce BCA protein assay kit (catalog No. 23227; Thermo Fisher Scientific, Waltham, MA, USA). Details of the Western blot procedure were reported previously by our group [9]. Briefly, protein samples were denatured by heating at 95 °C for 5 min before loading 20 µL protein into each lane of a 4–20% SDS-PAGE gel (catalog No. 4561094; Bio-Rad, Hercules, CA, USA). Reactions were run for 10 min at 180 V, and then for 45 to 60 min at 110 V. After activating a polyvinylidene fluoride membrane (catalog No. 1620261; Bio-Rad, Hercules, CA, USA) with methanol for 1 min, the protein sample was transferred to the membrane in a Trans-Blot SD Semi-Dry Electrophoretic Transfer Cell (catalog No. 170-3940; Bio-Rad, Hercules, CA, USA). Membranes were then blocked in 1× Tris-buffered saline (TBST) containing 5% nonfat milk for 2 h at room temperature. Membranes were then incubated in 1× TBST containing primary antibodies to total mTOR (mTORC1 and mTORC2), phospho-mTOR (Ser2448), AKT, phospho-AKT (Ser473), eukaryotic elongation factor 2 (eEF2), phospho-eEF2 (Thr56), solute carrier family 38 member 1 (SLC38A1), and BCKDK overnight at 4 °C; catalog number and dilution ratios are included in Supplemental Table S3. Membranes were then washed six times with 1× TBST and incubated with anti-rabbit horseradish peroxidase-conjugated secondary antibodies (catalog No. 7074S; dilution 1:800; Cell Signaling Technology, Danvers, MA, USA) for 1 h at room temperature. Subsequently, membranes were washed six times with 1× TBST and then incubated with enhanced chemiluminescence reagent (catalog No. 170-5060; Bio-Rad, Hercules, CA, USA) for 3 min in the dark prior to image acquisition. β-actin (catalog No. 4967S; Cell Signaling Technology, Danvers, MA, USA) was used as the internal control. Images were acquired using the ChemiDOC MP Imaging System (Bio-Rad, Hercules, CA, USA). The intensities of the bands were measured with Image-Pro Plus 6.0 software (Media Cybernetics, Rockville, MD, USA). Specific target protein band density values were normalized to β-actin density values. Representative blots with band size information are included in Figure S1.

### 2.4. Statistical Analysis

Statistical analysis was performed using the MIXED procedure in SAS v.9.4 (SAS Institute Inc., Cary, NC, USA). The fixed effect in the model was the ratio of Lys:Ile, Lys:Val, and Lys:Leu. The random effect was cow. Variables were assessed for normality of distribution using the Shapiro–Wilk test. Non-normally distributed data were log^2^-scale transformed to fit the normal distribution of residuals. Least squares means and standard errors were determined using the LSMEANS statement of SAS v.9.4 (SAS Institute Inc.) and were compared using Tukey. Significance was determined at *p* ≤ 0.05 and tendencies at *p* ≤ 0.10.

## 3. Results

**Figure 1:**
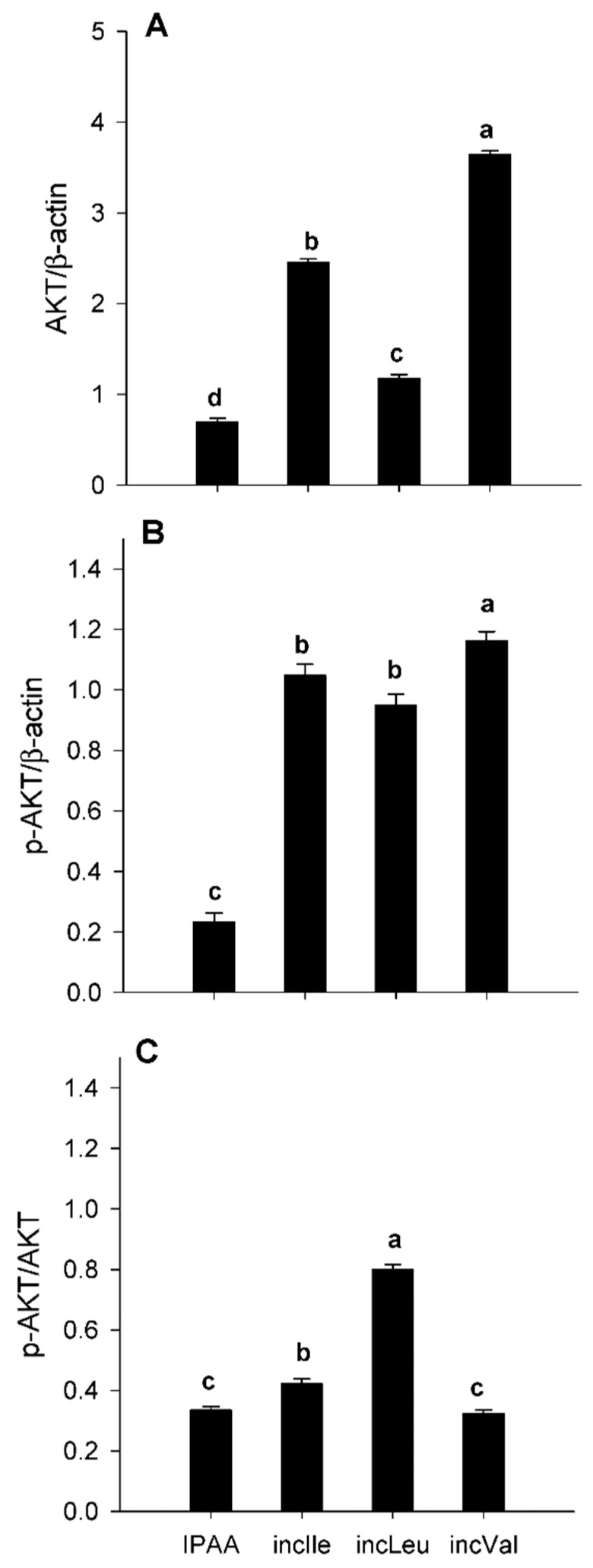
Protein abundance (relative to β-actin) of protein kinase B (AKT; total, panel (**A**)), p-AKT (active, panel (**B**)), and ratio of p-AKT/AKT (panel (**C**)) in s.c. adipose tissue cultured with different levels of Leu, Ile, or Val. Control media contained an ideal AA profile (IPAA; Lys:Met 2.9:1, Lys:Val 1.23:1; Lys:Ile 1.45:1; Lys:Leu 0.85:1). Treatment media was supplemented with greater amounts of Leu, Ile, or Val to achieve ratios of Lys:Leu 0.78:1 (incLeu), Lys:Ile 1.29:1(incIle), or Lys:Val 1.12:1 (incVal). Different letters indicate differences between treatments (*p* < 0.05) using the Tukey multiple comparison procedure in SAS v.9.4 (SAS Institute Inc., Cary, NC, USA). Data are LS means, *n* = 4 cows per group, ±pooled SEMs.

**Figure 2:**
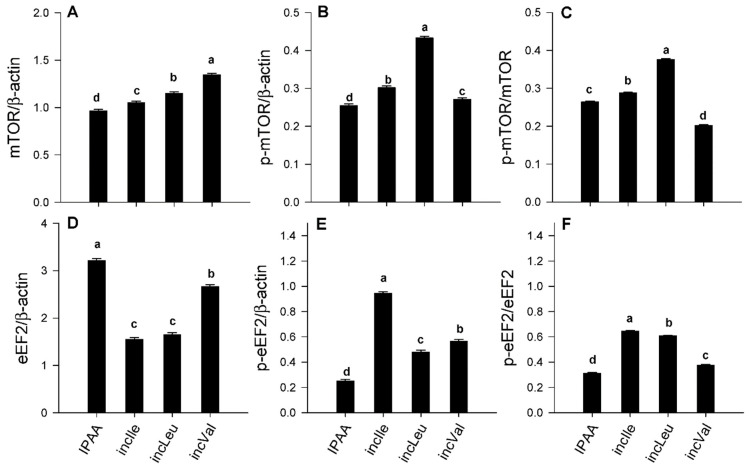
Protein abundance (relative to β-actin) of mechanistic target of rapamycin (mTOR; total, panel (**A**)), p-mTOR (active, panel (**B**)), ratio of p-mTOR/mTOR (panel (**C**)), eukaryotic elongation factor 2 (eEF2; total, panel (**D**)), p-eEF2 (active, panel (**E**)), and ratio of p-eEF2/eEF2 (panel (**F**)) in s.c. adipose tissue cultured with different levels of Leu, Ile, or Val. Control media contained an ideal AA profile (IPAA; Lys:Met 2.9:1, Lys:Val 1.23:1; Lys:Ile 1.45:1; Lys:Leu 0.85:1). Treatment media was supplemented with greater amounts of Leu, Ile, or Val to achieve ratios of Lys:Leu 0.78:1 (incLeu), Lys:Ile 1.29:1(incIle), or Lys:Val 1.12:1 (incVal). Different letters indicate differences between treatments (*p* < 0.05) using the Tukey multiple comparison procedure in SAS v.9.4 (SAS Institute Inc., Cary, NC, USA). Data are LS means, *n* = 4 cows per group, ±pooled SEMs.

Despite individual differences in abundance of AKT and p-AKT (Figure 1A,B), compared with IPAA, incLeu and incIle supplementation led to greater activation of AKT (p-AKT/total AKT) (*p* < 0.05, Figure 1C). Similarly, despite individual differences in mTOR and p-mTOR (Figure 2A,B), activity of mTOR (p-mTOR/total mTOR) had a more pronounced response with incLeu (*p* < 0.05, Figure 2C). It is noteworthy that incVal resulted in lower activation of mTOR (p-mTOR/total mTOR) compared with IPAA (*p* < 0.05, Figure 2C). 

**Figure 3:**
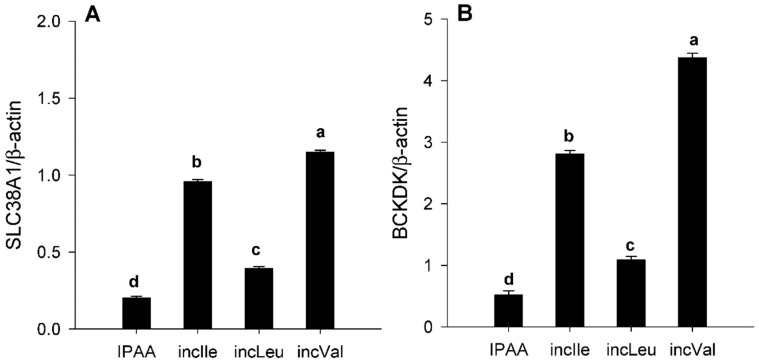
Protein abundance (relative to β-actin) of the Gln transporter SLC38A1 (panel (**A**)) and branched chain keto acid dehydrogenase kinase (BCKDK, panel (**B**)) in s.c. adipose tissue cultured with different levels of Leu, Ile, or Val. Control media contained an ideal AA profile (IPAA; Lys:Met 2.9:1, Lys:Val 1.23:1; Lys:Ile 1.45:1; Lys:Leu 0.85:1). Treatment media was supplemented with greater amounts of Leu, Ile, or Val to achieve ratios of Lys:Leu 0.78:1 (incLeu), Lys:Ile 1.29:1 (incIle), or Lys:Val 1.12:1 (incVal). Different letters indicate differences between treatments (*p* < 0.05) using the Tukey multiple comparison procedure in SAS v.9.4 (SAS Institute Inc., Cary, NC, USA). Data are LS means, *n* = 4 cows per group, ±pooled SEMs.

Despite individual changes in eEF2 and p-eEF2 abundance (Figure 2D,E) compaed with IPAA, supplementation of incLeu, incIle, and incVal led to greater activation of eEF2 (p-eEF2/total eEF2) (*p* < 0.05, Figure 2F). Compared with IPAA, protein abundance of SLC38A1 and BCKDK was greater with incLeu and greatest with incIle and incVal (*p* < 0.05, Figure 3A,B).

## 4. Discussion

It is well-established that SAT is an important insulin-sensitive tissue in dairy cows, e.g., phosphorylation of protein kinase B (AKT) responds to greater circulating glucose concentrations [13]. Overfeeding a high-starch/high-energy diet in the prepartum [14] or to dry/non-pregnant Holstein cows [15] upregulated lipogenic and insulin-responsive genes in SAT. Besides these well-established physiologic adaptations, dairy cow SAT is immune-responsive [16] and immune-responsive genes are expressed not only in SAT, but also in other fat depots of the cow [17]. These data underscored the complexity of mechanisms potentially controlling SAT function.

In non-ruminants, it is well-established that BCAA are not only building blocks for protein synthesis, but also key regulators of the mTOR signaling pathway [18]. Besides skeletal muscle, liver, and mammary cells, rodent studies have revealed that adipose tissue might play a role in modulating BCAA metabolism partly via changes in activity of BCAA catabolic enzymes such as BCAT2 and branched-chain α-keto acid dehydrogenase kinase (BCKDK) [19,20,21]. Alterations in the abundance of plasma membrane AA transporters and extracellular sensors of AA availability can also control BCAA metabolism, including signaling via mTORC1 [22]. Emerging evidence also indicates that adipose tissue might be a target organ for BCAA metabolism in dairy cows [8].

To the best of our knowledge, research on the effects of BCAA supply to dairy cows has mainly focused on the regulation of protein synthesis in mammary gland or isolated mammary cells [4,5,6]. Indeed, in vitro studies revealed that BCAA could promote protein synthesis via activation of the mTOR pathway [4,5]. It is noteworthy that human and rodent studies have provided evidence that increased circulating BCAA can predict metabolic disorders such as insulin resistance and diabetes [23]. Thus, exploring the effects of BCAA on mTOR and insulin signaling in SAT might provide new perspectives on the nutritional management of dairy cows during periods when they are most susceptible to metabolic disorders.

The mechanistic target of rapamycin is composed of two distinct complexes, mTORC1 and mTORC2 [24]. The former stimulates protein synthesis prior to contributing to cell growth and proliferation, and the latter mediates cell survival and proliferation as a function of AKT activation state [2]. The greater activation of AKT induced by incLeu and incIle supplementation in the present study suggested that these EAAs contribute (at least in vitro) to maintaining insulin signaling in SAT [9,13]. Eukaryotic translation elongation factor 2, a downstream target of the mTORC1 signaling pathway, controls protein synthesis [25]. Although, in the present study, we did not measure proliferation or apoptosis, the fact that incLeu or incIle led to activation of AKT (p-AKT/total AKT) and eEF2 (p-eEF2/total eEF2) led us to speculate that mTORC2 rather than mTORC1 might have been the primary branch responding to the supply of BCAA. Thus, these results suggest that greater activation of mTOR in SAT might play a more important role in controlling cell survival and proliferation rather than protein synthesis. 

At least in non-ruminants, the mTOR signaling pathway exerts some control on adipose biology and function via regulating aspects of lipid metabolism and adipokine synthesis/secretion [26]. For instance, adipocyte-specific *MTOR*-silencing in mice led to insulin resistance and inhibited adipocyte differentiation via the peroxisome proliferator-activated receptor γ signaling pathway [27]. In another study, silencing of eukaryotic translation initiation factor 2 alpha kinase 4 (a.k.a. general control nonderepressible 2) in mice led to reduced adipose tissue mass when fed a Leu-deficient diet [28]. The degradation of BCAA produces acetyl-CoA and succinyl-CoA, both of which are important intermediates in the TCA cycle and contribute carbon for lipogenesis in adipose tissue [29,30]. Together, these data agree with the fact that 3T3-L1 pre-adipocytes consume greater amounts of BCAA during differentiation [31]. From a mechanistic standpoint, the fact that BCAA, particularly Leu, can activate mTORC1 in mammalian cells [32] including adipose [33] led us to speculate about a direct effect of Leu on mTOR in the present study. Thus, the greater activation of mTOR (p-mTOR/total mTOR) in response to incLeu and incIle supplementation suggested that BCAA might play a dual role as regulators of mTOR and stimulators of lipogenesis, i.e., they could play dual functions in SAT.

Besides BCAA, Gln also plays an important role in regulating the mTOR signaling pathway, with several AA transporters (e.g., SLC38A1, solute carrier family 1 member 5, SLC1A5; solute carrier family 1 member 5, SLC7A5) being responsible for cellular Gln uptake or export [34]. For instance, SLC38A1, a neutral AA transporter, controls Gln transport into cells [35]. Inhibition of SLC1A5 prevents L-Gln uptake, resulting in a reduction in mTOR signaling; SLC7A5, a heterodimeric bidirectional antiporter, mediates the exchange of intracellular L-Gln for extracellular L-Leu [34]. Greater BCAA supply upregulated mRNA abundance of AA transporters in MAC-T cells [5]. Thus, we speculate that reduced SLC38A1 in response to incLeu might contribute to maintaining intracellular Leu and Gln homeostasis. 

In non-ruminant cells, BCKDK, a rate-limiting enzyme of BCAA catabolism, regulates intracellular concentrations of BCAA via inactivation and phosphorylation of the BCKD complex [36]. Thus, the greater protein abundance of BCKDK in response to incLeu, incIle, and incVal supplementation in the present study implies a greater intracellular availability of BCAA. Such a response also helps explain the greater activation of mTOR. The similar pattern of BCKDK and SLC38A1 suggested that Gln along with BCAA might potentially regulate mTOR signaling in bovine SAT. However, the exact mechanisms whereby Gln and BCAA interact in bovine SAT are unknown and merit further study.

Some limitations of the present study should be acknowledged. First, by design, this study narrowly focused on selected components of the mTOR, AA, and insulin signaling under basal conditions. Thus, we were unable to determine an effect of insulin per se. To address the link between BCAA supply and insulin signaling in SAT, future work could include an insulin challenge. Second, adipose tissue was obtained from late-lactation cows, which cannot reflect the responsiveness to BCAA in SAT obtained from peripartal cows. Third, the total supply of AA used can be considered supraphysiological and, although the level is similar to previous work with mammary cells and mammary tissue [4,5], it is unlikely that such levels would reach the SAT in vivo.

## 5. Conclusions

Overall, increased Leu or Ile supplementation contributes to greater activation of mTOR without impairing insulin signaling, which might be partly explained by increased AA transport and reduced BCAA catabolism in SAT. Because of the potential practical applications of enhancing post-ruminal supply of BCAA via feeding in rumen-protected form, in vivo studies are warranted. For example, abomasal infusions of BCAAs under negative energy balance would help assess the benefit of post-ruminal BCAAs on adipose function. Those data could help guide subsequent studies with periparturient cows.

## Figures and Tables

**Table 1:** Amino acid (AA) composition of the culture media with an ideal AA profile (IPAA; Lys:Ile ratio of 1.29:1; Lys:Val ratio of 1.12:1; Lys:Leu ratio of 0.78:1) and treatment media supplemented with greater amounts of Leu (incLeu), Ile (incIle), or Val (incVal) to alter ratios of Lys:Leu, Lys:Ile, or Lys:Val relative to IPAA.

Amino Acid	IPAA ^1^	IncLeu ^2^	incIle	incVal
L-Lys (µg/mL)	175 (1197 µmol/L)	175 (1197 µmol/L)	175 (1197 µmol/L)	175 (1197 µmol/L)
L-Met (µg/mL)	60 (402 µmol/L)	60 (402 µmol/L)	60 (402 µmol/L)	60 (402 µmol/L)
Lys:Met	2.9:1	2.9:1	2.9:1	2.9:1
L-Leu (µg/mL)	206 (1570 µmol/L)	225 (1570 µmol/L)	206 (1570 µmol/L)	206 (1570 µmol/L)
Lys:Leu	0.85:1	0.78:1	0.85:1	0.85:1
L-Ile (µg/mL)	121 (922 µmol/L)	121 (922 µmol/L)	136 (922 µmol/L)	121 (922 µmol/L)
Lys:Ile	1.45:1	1.45:1	1.29:1	1.45:1
L-Val (µg/mL)	142 (1212 µmol/L)	142 (1212 µmol/L)	142 (1212 µmol/L)	156 (1212 µmol/L)
Lys:Val (µg/mL)	1.23:1	1.23:1	1.23:1	1.12:1
L-Arg (µg/mL)	84 (482 µmol/L)	84 (482 µmol/L)	84 (482 µmol/L)	84 (482 µmol/L)
L-His (µg/mL)	74 (477 µmol/L)	74 (477 µmol/L)	74 (477 µmol/L)	74 (477 µmol/L)
L-Phe (µg/mL)	93 (563 µmol/L)	93 (563 µmol/L)	93 (563 µmol/L)	93 (563 µmol/L)
L-Thr (µg/mL)	97 (814 µmol/L)	97 (814 µmol/L)	97 (814 µmol/L)	97 (814 µmol/L)
L-Trp (µg/mL)	16 (78 µmol/L)	16 (78 µmol/L)	16 (78 µmol/L)	16 (78 µmol/L)
Total	1068 (7719 µmol/L)	1087 (7719 µmol/L)	1083 (7719 µmol/L)	1082 (7719 µmol/L)

^1^ IPAA = ideal AA profile, used as control medium. Ratios of essential AA are as follows: Lys:Met = 2.9, Lys:Thr = 1.8, Lys:His = 2.38, Lys:Val = 1.23, and Thr:Phe = 1.05 were based on NRC (2001) and our previous studies [Dong et al. (2018)]. ^2^ Composition of AA in the medium was prepared as described by Liang et al. (2021).

## Data Availability

The data reported in this manuscript are available upon reasonable request from the corresponding author (J.J.L.).

## Supplementary Materials

The following are available online at https://www.mdpi.com/article/10.3390/ani11092714/s1. Table S1: Ingredient composition of the lactation diet fed to cows; Table S2: Chemical composition and associated standard deviations for diets fed to cows; Table S3: Catalog number and source, dilution ratios, and target protein antibodies used in the present study; Table S4. Densitometry readings for individual proteins to the intensity of β-Actin; Figure S1: Representative blots with band size information.

## Abbreviations

AA	Amino acid
AKT	Protein kinase B
BCAT2	Branched-chain aminotransferase
BCKDK	Branched-chain alpha-ketoacid dehydrogenase kinase
BCAA	Branched-chain amino acids
eEF2	Eukaryotic elongation factor 2
EAA	Essential AA
Gln	Glutamine
incIle	Increased isoleucine
incLeu	Increased leucine
incVal	Increased valine
Ile	Isoleucine
IPAA	Ideal profile of AA
Leu	Leucine
Lys	Lysine
Met	Methionine
mTOR	Mechanistic target of rapamycin
mTORC1	Mechanistic target of rapamycin, complex 1
mTORC2	Mechanistic target of rapamycin, complex 2
NRC	National Research Council
SAT	Subcutaneous adipose tissue
SLC38A1	Na-coupled neutral amino acid transporter
Val	Valine

## References

[1] Yang X., Yang C., Farberman A., Rideout T.C., De Lange C.F.M., France J., Fan M.Z. (2008). The mammalian target of rapamycin-signaling pathway in regulating metabolism and growth^1,2^. J. Anim. Sci..

[2] Laplante M., Sabatini D.M. (2009). mTOR signaling at a glance. J. Cell Sci..

[3] Burgos S.A., Dai M., Cant J.P. (2010). Nutrient availability and lactogenic hormones regulate mammary protein synthesis through the mammalian target of rapamycin signaling pathway. J. Dairy Sci..

[4] Appuhamy J.A.D.R.N., Knoebel N.A., Nayananjalie W.A.D., Escobar J., Hanigan M.D. (2012). Isoleucine and Leucine Independently Regulate mTOR Signaling and Protein Synthesis in MAC-T Cells and Bovine Mammary Tissue Slices. J. Nutr..

[5] Dong X., Zhou Z., Wang L., Saremi B., Helmbrecht A., Wang Z., Loor J. (2018). Increasing the availability of threonine, isoleucine, valine, and leucine relative to lysine while maintaining an ideal ratio of lysine: Methionine alters mammary cellular metabolites, mammalian target of rapamycin signaling, and gene transcription. J. Dairy Sci..

[6] Appuhamy J., Knapp J., Becvar O., Escobar J., Hanigan M. (2011). Effects of jugular-infused lysine, methionine, and branched-chain amino acids on milk protein synthesis in high-producing dairy cows. J. Dairy Sci..

[7] Haffari M.H., Jahanbekam A., Sadri H., Schuh K., Dusel G., Prehn C., Adamski J., Koch C., Sauerwein H. (2019). Metabolomics meets machine learning: Longitudinal metabolite profiling in serum of normal versus overconditioned cows and pathway analysis. J. Dairy Sci..

[8] Webb L., Sadri H., Schuh K., Egert S., Stehle P., Meyer I., Koch C., Dusel G., Sauerwein H. (2020). Branched-chain amino acids: Abundance of their transporters and metabolizing enzymes in adipose tissue, skeletal muscle, and liver of dairy cows at high or normal body condition. J. Dairy Sci..

[9] Liang Y., Batistel F., Parys C., Loor J. (2019). Methionine supply during the periparturient period enhances insulin signaling, amino acid transporters, and mechanistic target of rapamycin pathway proteins in adipose tissue of Holstein cows. J. Dairy Sci..

[10] Minuti A., Bionaz M., Lopreiato V., Janovick N.A., Rodriguez-Zas S.L., Drackley J.K., Loor J.J. (2020). Prepartum dietary energy intake alters adipose tissue transcriptome profiles during the periparturient period in Holstein dairy cows. J. Anim. Sci. Biotechnol..

[11] National Research Council (2001). Nutrient Requirements of Dairy Cattle.

[12] Liang Y., Ma N., Coleman D.N., Liu F., Li Y., Ding H., Cardoso F.F., Parys C., Cardoso F.C., Loor J.J. (2021). Methionine and Arginine Supply Alters Abundance of Amino Acid, Insulin Signaling, and Glutathione Metabolism-Related Proteins in Bovine Subcutaneous Adipose Explants Challenged with N-Acetyl-D-sphingosine. Animals.

[13] Zachut M., Honig H., Striem S., Zick Y., Boura-Halfon S., Moallem U. (2013). Periparturient dairy cows do not exhibit hepatic insulin resistance, yet adipose-specific insulin resistance occurs in cows prone to high weight loss. J. Dairy Sci..

[14] Ji P., Osorio J., Drackley J., Loor J. (2012). Overfeeding a moderate energy diet prepartum does not impair bovine subcutaneous adipose tissue insulin signal transduction and induces marked changes in peripartal gene network expression. J. Dairy Sci..

[15] Ji P., Drackley J., Khan M., Loor J. (2014). Inflammation- and lipid metabolism-related gene network expression in visceral and subcutaneous adipose depots of Holstein cows. J. Dairy Sci..

[16] Mukesh M., Bionaz M., Graugnard D., Drackley J., Loor J. (2010). Adipose tissue depots of Holstein cows are immune responsive: Inflammatory gene expression in vitro. Domest. Anim. Endocrinol..

[17] Moisá S.J., Ji P., Drackley J.K., Rodriguez-Zas S.L., Loor J.J. (2017). Transcriptional changes in mesenteric and subcutaneous adipose tissue from Holstein cows in response to plane of dietary energy. J. Anim. Sci. Biotechnol..

[18] Zhang S., Zeng X., Ren M., Mao X., Qiao S. (2017). Novel metabolic and physiological functions of branched chain amino acids: A review. J. Anim. Sci. Biotechnol..

[19] Herman M.A., She P., Peroni O.D., Lynch C.J., Kahn B.B. (2010). Adipose Tissue Branched Chain Amino Acid (BCAA) Metabolism Modulates Circulating BCAA Levels. J. Biol. Chem..

[20] Blanchard P.-G., Moreira R., Castro É., Caron A., Côté M., Andrade M.L., Oliveira T.E., Ortiz-Silva M., Peixoto Á.S., Dias F.A. (2018). PPARγ is a major regulator of branched-chain amino acid blood levels and catabolism in white and brown adipose tissues. Metabolism.

[21] Yoneshiro T., Wang Q., Tajima K., Matsushita M., Maki H., Igarashi K., Dai Z., White P.J., McGarrah R.W., Ilkayeva O.R. (2019). BCAA catabolism in brown fat controls energy homeostasis through SLC25A44. Nat. Cell Biol..

[22] Zhou Y., Ren J., Song T., Peng J., Wei H. (2016). Methionine Regulates mTORC1 via the T1R1/T1R3-PLCβ-Ca^2+^-ERK1/2 Signal Transduction Process in C2C12 Cells. Int. J. Mol. Sci..

[23] McCormack S.E., Shaham O., McCarthy M.A., Deik A.A., Wang T., Gerszten R.E., Clish C.B., Mootha V.K., Grinspoon S.K., Fleischman A. (2013). Circulating branched-chain amino acid concentrations are associated with obesity and future insulin resistance in children and adolescents. Pediatr. Obes..

[24] Wullschleger S., Loewith R., Hall M.N. (2006). TOR Signaling in Growth and Metabolism. Cell.

[25] Kaul G., Pattan G., Rafeequi T. (2011). Eukaryotic elongation factor-2 (eEF2): Its regulation and peptide chain elongation. Cell Biochem. Funct..

[26] Cai H., Dong L.Q., Liu F. (2016). Recent Advances in Adipose mTOR Signaling and Function: Therapeutic Prospects. Trends Pharmacol. Sci..

[27] Shan T., Zhang P., Jiang Q., Xiong Y., Wang Y., Kuang S. (2016). Adipocyte-specific deletion of mTOR inhibits adipose tissue development and causes insulin resistance in mice. Diabetologia.

[28] Guo F., Cavener D.R. (2007). The GCN2 eIF2α Kinase Regulates Fatty-Acid Homeostasis in the Liver during Deprivation of an Essential Amino Acid. Cell Metab..

[29] Sears D.D., Hsiao G., Hsiao A., Yu J.G., Courtney C.H., Ofrecio J.M., Chapman J., Subramaniam S. (2009). Mechanisms of human insulin resistance and thiazolidinedione-mediated insulin sensitization. Proc. Natl. Acad. Sci. USA.

[30] Song Z., Xiaoli A.M., Yang F. (2018). Regulation and Metabolic Significance of De Novo Lipogenesis in Adipose Tissues. Nutrients.

[31] Green C., Wallace M., Divakaruni A.S., Phillips S.A., Murphy A.N., Ciaraldi T.P., Metallo C.M. (2016). Branched-chain amino acid catabolism fuels adipocyte differentiation and lipogenesis. Nat. Chem. Biol..

[32] Lynch C.J., Adams S. (2014). Branched-chain amino acids in metabolic signalling and insulin resistance. Nat. Rev. Endocrinol..

[33] Lynch C.J., Patson B.J., Anthony J., Vaval A., Jefferson L.S., Vary T.C. (2002). Leucine is a direct-acting nutrient signal that regulates protein synthesis in adipose tissue. Am. J. Physiol. Metab..

[34] Nicklin P., Bergman P., Zhang B., Triantafellow E., Wang H., Nyfeler B., Yang H., Hild M., Kung C., Wilson C. (2009). Bidirectional Transport of Amino Acids Regulates mTOR and Autophagy. Cell.

[35] MacKenzie B., Erickson J.D. (2004). Sodium-coupled neutral amino acid (System N/A) transporters of the SLC38 gene family. Pflüg. Arch.-Eur. J. Physiol..

[36] Shimomura Y., Obayashi M., Murakami T., Harris R.A. (2001). Regulation of branched-chain amino acid catabolism: Nutritional and hormonal regulation of activity and expression of the branched-chain α-keto acid dehydrogenase kinase. Curr. Opin. Clin. Nutr. Metab. Care.

