# Supplementary material for: Branched-Chain Amino Acid Supplementation Alters the Abundance of Mechanistic Target of Rapamycin and Insulin Signaling Proteins in Subcutaneous Adipose Explants from Lactating Holstein Cows
Source: Animals (Basel). 2021 Sep 17;11(9):2714. doi: 10.3390/ani11092714 (PMC8470689; doi:10.3390/ani11092714)
Supplement: Supplementary file 1 [file animals-11-02714-s001.zip › animals-1324144-supplementary.pdf]

Supplemental Table S1. Ingredient composition of the lactation diet fed to all cows during the experimental period.

Ingredient	% of DM
Corn silage	40.2
Alfalfa hay	18.4
Dry ground corn grain	17.3
Corn gluten feed pellets	7.9
Canola meal expelled	5.6
Vitamin and mineral mix[1]	4.4
Molasses	3.4
Rumen inert fat[2]	1.5
Blood meal[3]	0.4
Rumen-protected lysine[4]	0.4
Urea 281 CP	0.4
Rumen-protected methionine[5]	0.1

[1]Mineral and vitamin mix was formulated to contain 12.51% Ca, 14.06% Na, 9.60% Cl, 3.18% Mg, 6.48% K, 0.19% S, 26.93 mg/kg Co, 301.01 mg/kg of Cu, 40.22 mg/kg of I, 678.25 mg/kg Fe, 1,519.35 mg/kg Mn, 8.62 mg/kg Se, 4.47 mg/kg of organic Se, 1621.05 mg/kg of Zn, 43.34 kIU/kg Vitamin A, 10.89 kIU/kg of Vitamin D_3, 466.41 IU/kg of Vitamin E, 4.23 mg/kg of biotin, 46.65 mg/kg of thiamine, and 0.35 g/kg of monensin (Rumensin, Elanco, Greenfield, IN)
[2]Energy Booster 100 (Milk Specialties Global, Eden Prairie, MN)
[3]ProVAAl AADvantage (Perdue AgriBusiness, Salisbury, MD)
[4]Ajipro-L Generation 3 (Ajinomoto Heartland, Inc., Chicago, IL)
[5]Smartamine M (Adisseo, Alpharetta, GA).

Supplemental Table S2. Mean chemical composition and associated standard deviations for diet fed to all cows throughout the experimental period (TMR samples n=7).

Item	Mean	SD
DM, %	49.83	3.74
CP, % of DM	15.74	0.75
ADF, % of DM	20.21	1.92
NDF, % of DM	30.66	1.80
Lignin, % of DM	3.74	0.70
NFC, % of DM	41.81	2.22
Starch, % of DM	27.19	3.23
Crude fat, % of DM	4.30	0.32
Ash, % of DM	7.44	0.90
NE_L, Mcal/kg of DM	0.77	0.02
Ca, % of DM	0.82	0.13
P, % of DM	0.44	0.03
Mg, % of DM	0.29	0.02
K, % of DM	1.59	0.14
Na, % of DM	0.56	0.08
S, % of DM	0.25	0.01
Fe, ppm	260	81
Zn, ppm	101.86	14.44
Cu, ppm	13.57	1.72
Mn, ppm	76.14	7.08
Mo, ppm	1.03	0.36

Supplemental Table S3. Antibody symbol, catalog number, company, dilution ratio, and antibody name for antibodies measured in dairy cow subcutaneous adipose tissue.

Antibody	Catalog Number	Company	Dilution ratio	Antibody Name
mTOR	2972S	Cell Signaling Technology	1:500	Mechanistic target of rapamycin
Phospho-mTOR(Ser2448)	2971S	Cell Signaling Technology	1:250	Phosphorylated mechanistic target of rapamycin
AKT	9272S	Cell Signaling Technology	1:500	Protein kinase B
Phospho-AKT(Ser473)	9271S	Cell Signaling Technology	1:250	Phosphorylated protein kinase B
SLC38A1	ab60145	Abcam	1:250	Solute carrier family 38 member 1
BCKDK	ab151297	Abcam	1:500	Branched-chain α-keto acid dehydrogenase kinase
eEF2	2332s	Cell Signaling Technology	1:500	Eukaryotic elongation factor 2
Phospho-eEF2 (Thr56)	2331s	Cell Signaling Technology	1:250	Phosphorylated eukaryotic elongation factor 2

Supplemental Table S4. Densitometry readings for individual proteins to the intensity of β-Actin.

	mTOR	p-mTOR	eEF2	p-eEF2	Akt	p-Akt	SCL38A1	BCKDK
IPAA	0.2491	0.2545	3.2158	0.2491	0.6980	0.2329	0.2021	0.5175
Ile	0.9441	0.3020	1.5503	0.9441	2.4510	1.0481	0.9583	2.8089
Leu	0.4796	0.4327	1.6505	0.4796	1.1784	0.9497	0.3933	1.0871
Val	0.5652	0.2709	2.6649	0.5652	3.6435	1.1634	1.1496	4.3745

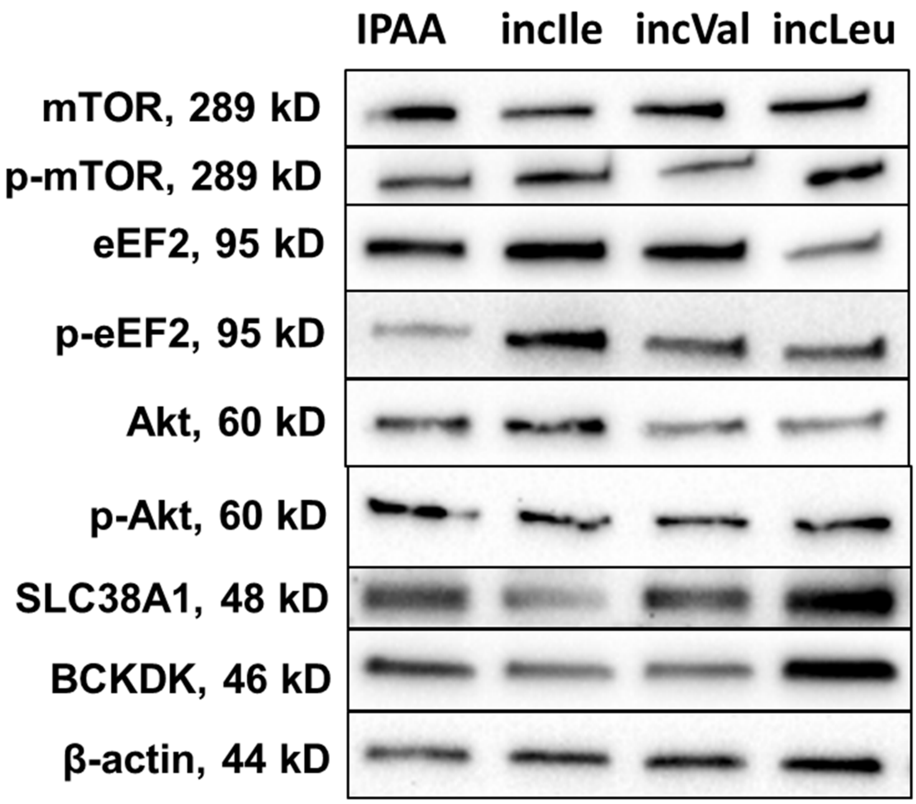

Supplemental Figure S1. Representative blots with band size information. Original blot images from the present study were inadvertently lost in the month of March 2021 when Animal Sciences departmental computers were affected by a virus that prevented the proper launching of applications (e.g. Chrome, Outlook). Fixing this issue required Windows to be reinstalled in all departmental computers prior to the reimaging process. During this process, however, only the files and profiles of the most-recent users on a given computer were backed up. Since the lead author of the manuscript (YL) had not logged into the laboratory computer that contained blot images for months, his profile was not backed up during the reimaging process.
